# Navigating a fragile capability: meanings of encountering patients in a suicidal process from the perspective of ambulance clinicians

**DOI:** 10.1080/17482631.2026.2717806

**Published:** 2026-08-12

**Authors:** Staffan Hammarbäck, Anders Bremer, Mats Holmberg, Lena Wiklund Gustin

**Affiliations:** a Linnaeus University, Faculty of Health and Life Sciences, Växjö, Sweden; b Linnaeus University, Centre of Interprofessional Collaboration within Emergency care (CICE), Växjö, Sweden; c Region Sörmland, Department of Ambulance Service, Katrineholm, Sweden; d Centre for Clinical Research Sörmland, Uppsala University, Eskilstuna, Sweden; e Department of Health Sciences, Innovation and Design, Mälardalen University, Eskilstuna/Västerås, Sweden; f UiT/The Arctic University of Norway, Department of Health and Care Sciences, Campus Narvik, Narvik, Norway

**Keywords:** Ambulance clinicians, phenomenological hermeneutical, prehospital care, professional identity, suicidal process, suicide prevention

## Abstract

**Purpose:**

To illuminate the meanings of encountering patients in a suicidal process from the perspective of ambulance clinicians.

**Methods:**

Narrative interviews were conducted with eighteen ambulance clinicians in Sweden. Data were analyzed using a phenomenological hermeneutical approach inspired by the philosophy of Ricœur.

**Results:**

The main theme, “Navigating a fragile capability,” reveals a profound tension within professional identity. Traditionally anchored in technical agency, this identity is challenged by a movement toward vulnerability and shared humanity. Capability in these encounters is defined not only by medical problem-solving but by the courage to remain present during existential crises. Entering the patient’s narrative imposes an extensive responsibility, where experiences of indecisiveness and powerlessness emerge as expressions of moral sensitivity rather than professional inadequacy.

**Conclusions:**

Encountering suicidal patients reveals professional capability as a fragile construct dependent on reciprocity, requiring a shift from technical agency toward an ethical presence when medical protocols reach their limit. By validating *being with* as a clinically meaningful component of care, the encounter moves beyond procedural care toward a meaningful ethical aim. Practically, professional standards must expand beyond technical management to integrate relational competence and existential care as core elements of prehospital practice.

## Background

Suicide is a critical public health challenge, accounting for over 700,000 deaths annually (World Health Organisation, 2021). Beyond its global statistical impact, suicide represents a profound personal tragedy that resonates through families and communities (Knipe et al., 2022). It does not stem from a single cause but arises from a complex interplay of factors across the lifespan. Among the most prominent risk factors are previous suicide attempts and mental illness (Turecki & Brent, 2016).

Drawing on Wasserman (2016), the suicidal process is conceptualised in this study as a continuous and fluctuating progression, stretching from initial suicidal ideation and communication to planning, attempts, and suicide death. At moments when mental suffering becomes unbearable, suicide may appear as the only perceived means to escape. Suicidal suffering is described as a total existential crisis where emotional pain is deeply intertwined with a sense of internal disintegration (Marcinkevičiūtė et al., 2024). From the perspective of those with lived experience, such a crisis represents an existential and relational collapse rather than a mere collection of clinical symptoms (Roennfeldt et al., 2024). This collapse implies that the individual’s everyday ways of understanding the world and themselves have ceased to function, leading to a profound sense of being lost. While most individuals with suicidal ideation do not attempt suicide, some develop a capability to transition from ideation to action (Bayliss et al., 2025). This progression involves an acquired capability and a heightened sense of agency, a paradoxical attempt to reclaim control in a state of powerlessness. Yet, even in moments preceding an action, a fundamental ambivalence often persists between acting and the hope of interruption (Larsen and Hawton, 2025). For the individual in a suicidal process, the quality of the immediate encounter with healthcare providers is paramount (Shamsaei et al., 2020). An empathetic encounter, characterised by the feeling of being truly seen and understood, can serve as a pivotal turning point for regaining hope.

As most individuals have contact with healthcare services before a suicide, emergency care settings constitute a critical juncture for suicide prevention (Ahmedani et al., 2019; Shin et al., 2021). Ambulance clinicians increasingly serve as a primary point of contact yet report limited confidence and preparedness when encountering patients in a suicidal process (Rees et al., 2018; Romeu et al., 2023). They describe a sense of apprehension regarding risk management and professional liability, compounded by the challenge of navigating the intersection of physical and mental health. This often results in reliance on hospital transport when referral options are limited. With a professional identity rooted in the management of physical trauma and medical emergencies, ambulance clinicians frequently perceive underlying mental illness as beyond their professional scope, constrained by limited training and the complexity of suicidal behaviour (Rees et al., 2015). These experiences are further shaped by time pressure and a perceived lack of viable prehospital treatment options. Furthermore, ambulance clinicians describe differences in their ways of understanding their responsibility in these encounters, a conception influenced by factors such as fatigue, collegial support, and the functioning of the healthcare system as a whole (Hammarbäck et al., 2023).

Despite the recognised challenges of these encounters, research has predominantly focused on procedural barriers and staff attitudes. There remains a significant gap in knowledge regarding the lived experience and the existential dimensions of the encounter itself. To move beyond clinical descriptions and foster a deeper understanding of the relationships that unfold in these crises, it is essential to explore the underlying meanings these encounters hold for the clinicians. Therefore, the aim of this study was to illuminate the meanings of encountering patients in a suicidal process from the perspective of ambulance clinicians.

## Research design

The phenomenon under study was *the meanings of encountering patients in a suicidal process as an ambulance clinician*. To illuminate the meanings of these encounters, a phenomenological hermeneutical approach was chosen, as described by Lindseth and Norberg (2004, 2022). This method is particularly well-suited for healthcare research as it allows for a movement from the clinicians’ lived experiences, expressed through narratives, to an interpretation of the essential meanings of the phenomenon. By bridging phenomenological focus on the lifeworld with hermeneutical emphasis on interpretation, the method provides a framework for uncovering dimensions of care that often remain unspoken in clinical practice. This inductive design is inspired by the phenomenology of Husserl (2014) and the hermeneutics of Ricœur (1991, 1976). The methodology relies on understanding lifeworld phenomena through narratives of lived experience. Within this methodology, the transcribed interviews are considered as a single text with the potential to open up a world of its own (Ricœur, 1991). During the analysis, the focus remains on the text itself rather than on the individual participants. Hermeneutic interpretation seeks both to explain the structure of the text and to uncover the meanings within it. To ensure rigorous and transparent reporting, this study adheres to the Standards for Reporting Qualitative Research (SRQR) guidelines (O’Brien et al., 2014).

### Participants and research context

A criterion-based volunteer sampling strategy across four regions in Sweden was utilised to select participants who could provide rich, information-dense narratives regarding the phenomenon. The inclusion criteria required participants to be employed as ambulance clinicians with a minimum of 1 year of professional experience in the ambulance service. No specific exclusion criteria were applied.

Recruitment was conducted across four regions in Sweden, utilising two different approaches adapted to regional organisational structures and practical feasibility. In two of the regions, the first author visited ambulance stations in person to inform the clinicians about the study, after which interested clinicians signed up to participate. In the remaining two regions, unit managers distributed written information about the study to the clinicians via email, and interested participants then contacted the first author directly by email to request further details. Because station visits and manager-distributed emails served as open recruitment routes, the total number of clinicians exposed to the invitation remains unknown. Following the provision of additional information, a single interested clinician chose to decline participation.

In accordance with the phenomenological hermeneutical methodology described by Lindseth and Norberg (2004, 2022), the transcribed interviews are treated as a single text through which essential meanings are disclosed. Sample size adequacy was evaluated using the concept of information power, considering the focused study aim, high sample specificity, theoretical foundation, dialogue quality, and analysis strategy (Malterud et al., 2016). Eighteen narrative interviews represent a substantial sample size within this design, producing an extensive text material that captured rich variations in lived experiences across four regions, professional roles, and levels of clinical experience. This comprehensive textual corpus provided a robust foundation to navigate the hermeneutic arch between the parts and the whole. Thematic saturation aimed at code exhaustion was neither sought nor considered methodologically appropriate for this interpretive design.

A total of eighteen ambulance clinicians (11 men, 7 women; aged 27–62 years) were recruited from four regions in Sweden. Their professional experience in the ambulance service ranged from 3 to 33 years. The participants comprised two emergency medical technicians, four registered nurses, and twelve registered nurses with specialist nurse education (e.g., anaesthesia care or prehospital emergency care). Three participants had prior experience in psychiatric care.

In the Swedish context, the ambulance service is nurse-led, requiring at least one crew member to be a registered nurse (Sjölin et al., 2019). While each region is responsible for its own ambulance organisation, equipment and specialised units vary. For instance, some urban areas utilise psychiatric emergency response units, whereas rural areas rely on standard ambulance crews for mental health crises (Bouveng et al., 2017; Lindström et al., 2020). Furthermore, the legal framework regarding involuntary care differs internationally (Roberts and Masters, 2025). In the Swedish healthcare system, the Health and Medical Services Act (Hälso- och sjukvårdslag, SFS 2017:30) stipulates that care must be of high quality and provided with respect for patient autonomy and dignity. However, a professional dilemma arises when a patient in a suicidal process refuses life-saving intervention. While the Compulsory Psychiatric Care Act (Lag om psykiatrisk tvångsvård, SFS 1991:1128) regulates involuntary treatment, it provides no specific mandate for ambulance clinicians to use physical restraint; such powers are legally reserved for the police, and medical certificates for compulsory care are issued by physicians. Consequently, in acute situations where clinicians must act against a patient’s will to prevent self-harm, they are forced to rely on the principle of necessity in the Swedish Penal Code (Brottsbalk, SFS 1962:700**)**, a provision applicable to all citizens rather than a professional clinical protocol. This legal framework necessitates close interagency collaboration, where police and ambulance services must cooperate to manage the balance between medical needs and physical safety (Nilsson et al., 2017). However, this dependence may also complicate immediate risk management and place ambulance clinicians in a position where they must manage high-risk situations without the legal authority to employ physical or pharmacological restraints.

### Data collection

Individual, audio-recorded, narrative interviews (Brinkmann & Kvale, 2015) were conducted between September and October 2024. The interviews were conducted in Swedish by the first author, who is an experienced ambulance clinician. The first author has previously conducted three qualitative interview studies, including one utilising a phenomenological hermeneutical approach. The co-authors provided extensive methodological expertise and oversight throughout the process. No prior relationship existed between the interviewer and the participants. Although four participants were employed within the same healthcare region as the first author, they were stationed at different locations and shared no prior collegial relationship.

No pilot interviews were undertaken. The narrative approach was chosen to encourage participants to share their lived experiences in the form of stories, thereby making meanings accessible for interpretation through the act of narration. The interviews began with the question: “*Can you tell me about an assignment where you encountered a patient in a suicidal process*?” To deepen the narration and capture lived experiences, follow-up questions such as “*How is it to you, when…?*” and “*What does…mean to you?”* were used. The interviews were conducted face to face at the participants’ workplace (*n* = 13), in the participants’ homes (*n* = 2) or via Zoom (*n* = 3) and lasted between 22 and 101 minutes (mean 48 minutes, median 42 minutes). Regarding the interviews conducted via Zoom, no noticeable differences in rapport, emotional connection, or narrative depth were observed compared to the face-to-face encounters.

All interviews were transcribed verbatim by the first author and subsequently verified against the original audio recordings to ensure accuracy. The participant quotations were kept and analysed in their original Swedish language throughout the structural analysis. Following the completion of the structural analysis, the selected quotations were translated into English by the first author prior to the comprehensive understanding. All authors reviewed the quotations in both Swedish and English during the manuscript preparation to ensure that the meanings were accurately reflected.

### Data analysis

According to Ricœu (1991), the interpretation of a text aims to understand the meaning of the utterance within the text and involves the dialectic of understanding and explanation. There is a movement between the understanding that the text opens up to the reader and the explanation given by the decontextualization in the structural analysis. Interpretation consists of a dialectical movement between distanciation, where the text is viewed as an independent discourse detached from its author, and appropriation, where the meaning of the text is integrated into one’s own understanding to open up new possibilities of being in the world (Wiklund et al., 2002).

The analysis followed an inductive approach based on the methodology of Lindseth and Norberg (2004, 2022) and the process includes naïve understanding, structural analysis, and comprehensive understanding. This process is conceptualised as a hermeneutic arch, where the movement between the parts and the whole generates a non-linear path toward deeper insight (Ricœur, 1991). Throughout this process, the text was treated as an autonomous entity through a process of distanciation, allowing the researchers to move beyond the immediate clinical context to identify underlying patterns. Initially, a n*aïve reading* was performed to acquire a general sense of the whole, striving to transition from a natural to a phenomenological attitude (Lindseth & Norberg, 2022). This resulted in a *naïve understanding,* serving as a first conjecture.

The entire analysis process was performed manually by the first author using word processing software over a period of several months. Specifically, the first author identified and condensed all meaning units across the dataset. All co-authors had access to the full transcripts and acted as critical evaluators throughout the process, continuously reviewing analytic summaries, condensations, and thematic formulations in relation to the narrative data during recurring collaborative meetings to ensure analytical rigour. This collaborative process spanned from the identification of the first meaning units to the formulation of the final findings. The authors held recurring digital meetings to discuss the progression of the analysis and the movement between the analytical steps. Furthermore, draughts of the analysis were continuously shared through email and reviewed by the co-authors. Any interpretive disagreements or alternative perspectives regarding the formulations of the themes were resolved through critical dialogue to achieve the most plausible interpretation.

In the subsequent *structural analysis*, the movement along the hermeneutic arch continued as meaning units were identified, condensed, and organised into subthemes and themes. Examples of the structural analysis are presented in Table I, where four rows were deliberately selected as illustrative examples to depict the analytic pathway across both comprehensive themes. This stage represented the methodological distanciation where the text was explained through its parts. In the final stage of the *comprehensive understanding*, the analysis moved toward re-contextualisation. Here, the naïve understanding and the structural findings were synthesised and reflected upon in the light of the ontology of *the capable human being* by Ricœur (2005, 1992). This dialectical movement allowed the main theme and the chosen theory to interact, expanding the understanding of the phenomenon beyond the initial reading, and achieving appropriation through the realisation of a perceived new meaning of the lived experience. The theoretical framework of the ontology of Ricœur regarding the capable human being was not used deductively to guide the initial structural analysis. Instead, in line with the phenomenological hermeneutical tradition described by Lindseth and Norberg (2004, 2022), it was selected after the structural analysis to illuminate and re-contextualise the findings at the level of comprehensive understanding. In accordance with the non-linear nature of the hermeneutic arch, selecting this theoretical lens initiated an iterative dialectic between the empirical themes and the concepts of Ricœur, which contributed to refining the final thematic formulations. Prior to selecting the framework of Ricœur, alternative interpretations, specifically the philosophy of Buber regarding the interhuman and the *I and Thou relationship*, were deliberated within the research team. However, the ontology of Ricœur was ultimately chosen as it provided a more comprehensive framework for capturing not only the relational encounter, but also the profound tension between clinicians’ traditional agency of *I can* and their existential vulnerability when protocols fall silent.

In accordance with the phenomenological hermeneutical tradition, the researchers’ pre-understanding was viewed not as a bias to be eliminated, but as a necessary condition for interpretation (Lindseth & Norberg, 2004, 2022). In this study, three of the authors have extensive clinical experience in ambulance care, while the fourth author has expertise in the psychiatric context. This combined preunderstanding was essential for being sensitive to the nuances of the participants’ narratives. To ensure that this professional background did not overshadow the participants’ voices, the analysis followed the structural steps in the method, creating a dialectic distance where the textual meaning could challenge the researchers’ assumptions (Ricœur, 1991).

Reflexivity was maintained through continuous critical dialogues within the research team, where emerging interpretations were constantly weighed against the original interview data to ensure they remained grounded in the participants’ narratives. Rather than utilising formal pre-analysis bracketing protocols, the authors relied on regular reflexive discussions during the iterative writing phases to challenge their internalised professional norms. For instance, the researchers were surprised by how frequently participants explicitly apologised for their emotional reactions during the encounters by stating that they were only human. This unexpected finding challenged the researchers’ clinical pre-understanding regarding the rigidity of the professional identity in ambulance care. It forced a critical re-evaluation of how deeply the expectation of absolute emotional detachment is embedded in the culture of prehospital services, thereby ensuring that the analysis captured the tension between the professional role and shared humanity.

**Table I.** Examples from the structural analysis.

Meaning unit	Condensation	Subtheme	Theme
*I felt a bit stressed there because, firstly, I have a patient who is actively trying to self-harm—that is one aspect that is stressing me. Secondly, how am I supposed to handle this? What am I allowed to do? Can I restrain her in this case? Am I allowed to deprive this patient of her liberty? I must be allowed to do that somehow… But how do I do this without feeling like a damn perpetrator, grabbing a girl like that?*	Feeling moral distress and legal uncertainty regarding the use of physical restraint, conflicting with professional duty and the desire for ethical integrity.	Balancing professional composure against internal fragility	*****Being vulnerable under the burden of responsibility*****
*When you end up in a situation like that and are the one who has to solve it... it’s just the helplessness you feel right there in what you can do for them that makes you dislike this type of assignment. Out on the run, I don’t feel like I’m doing an adequate job to help; I lack something in my toolbox, in my knowledge, to actually help!*	Experiencing a profound sense of helplessness and professional insufficiency during the crisis assignment, feeling that the lack of clinical tools prevents adequate help from being provided to the patient.	Being professionally and existentially powerless	*****Being vulnerable under the burden of responsibility*****
*I just felt a huge sense of relief when it happened, that the person in question began to emerge from some kind of—I don’t know what kind of world she was in, because it was like she was completely isolated in her own world. And then, just like a click, it opened up and she started talking to us and we connected. That situation, right then and there, was such a relief.*	Experiencing a profound sense of relief when a communicative breakthrough occurs, transforming the patient’s isolation into a shared interpersonal connection.	Being let in	*****Bridging isolation through a shared humanity*****
*I actually saw him afterwards... he sat and ate in the dining room at a sheltered housing facility... he sat with that group there and ate, and it was just nice, like yeah, okay, you’re alive, it was nice to see... and he nodded to me as if he recognised me, and it was just really good to see that he’s still alive*	Experiencing a sense of emotional relief and confirmation when unexpectedly seeing a former patient alive and socialising, validated through a mutual nod of recognition	Becoming part of each other’s narratives	*****Bridging isolation through a shared humanity*****

## Ethical considerations

The study was conducted in accordance with the ethical principles of the Declaration of Helsinki and approved by the Swedish Ethical Review Authority (Ref. Nos. 2019-03774 and 2024-02178). Ethical challenges in this study mainly concerned the sensitive nature of suicidality and the risk of evoking moral distress or painful memories during the narrative interviews. To address these concerns, several measures were taken. Participants received both written and oral information about the study and their right to withdraw at any time without explanation. Written informed consent was obtained prior to each interview. Given the sensitive nature of encountering patients in a suicidal process, the interviews were conducted with awareness of the potential for emotional distress among the participants (Dickson-Swift et al., 2007). While the participants were experienced clinicians, discussing these encounters can trigger reflections on emotionally demanding situations. To address this, the first author, who conducted the interviews and has clinical experience in ambulance care, remained attentive to the participants’ reactions throughout the interview process. Furthermore, time was allocated for informal reflection after each interview to allow participants to conclude the session in a professional and secure manner. The participants also had access to peer support and occupational health services through their employers.

Prior to the interviews, participants were explicitly informed that any details potentially identifying themselves, colleagues, patients, or next of kin would be de-identified. This instruction also extended to specific geographic locations and addresses. To encourage unconstrained storytelling and allow participants to freely narrate their lived experiences, clinicians were not instructed to omit identifying details during the interviews. Instead, all names, specific geographic locations, addresses, and organisational markers were systematically anonymized during transcription. Furthermore, distinctive or highly specific case details were generalised prior to selecting final quotations to safeguard third-party confidentiality. Consequently, the data analysis was conducted on a text completely stripped of any information that could enable identification. Participants were referred to by code numbers, with the code key only accessible to the first author.

Although the focus of the study was on the clinicians’ experiences, the narratives inherently involved patients in vulnerable situations. This was handled with the utmost respect for the patients’ dignity, ensuring that the focus remained strictly on the professional encounter and the meanings of the experiences, rather than the clinical details of individual cases.

## Findings

### Naïve understanding

Encountering patients in a suicidal process means being indecisive where established working procedures are insufficient. It implies insecurity, arising from a lack of conventional feedback to confirm the appropriateness of one’s actions or words. Thus, the encounter means being simultaneously powerless and in a position of power. A high regard for safety means that clinical patient assessment becomes secondary to the continuous evaluation of the environment for hazards and threats. Balancing the responsibility to provide care against the physical safety of oneself and colleagues involves managing conflicting emotions and vulnerability. The encounter means assuming a level of responsibility that exceeds experienced competence. It implies working under the expectation of professional mastery while being under-equipped and finding oneself in the silence of protocols. Furthermore, the encounter means facing the patient without adequate tools, reflecting an inability to provide meaningful help. When patients are unresponsive or reject intervention, it means that the professional role is profoundly challenged. Fearing that one might upset the patient or trigger aggressive or suicidal behaviour means accepting silence as the status quo. However, the encounter also implies the possibility of being meaningful. For instance, when a patient expresses gratitude or when the act of taking time is perceived to have offered relief, the encounter means a moment of genuine human connection.

### Structural analysis

The structural analysis resulted in the main theme *Navigating a fragile capability* which is comprised of two themes and six subthemes (see Table II).

**Table II.** Main theme, themes and subthemes.

Main theme	Navigating a fragile capability	
**Themes**	*****Being vulnerable under the burden of responsibility*****	*****Bridging isolation through a shared humanity*****
**Subthemes**	*Being professionally and existentially powerless*	*Being let in*
	*Balancing professional composure against internal fragility*	*Embodying solicitude through presence*
	*Being indecisive in the void of protocols*	*Becoming part of each other’s narratives*

The main theme represents the overarching meaning of the phenomenon. It is supported by two comprehensive themes that capture the tension between vulnerability and shared humanity, which are further specified and grounded by the six subthemes.

### Navigating a fragile capability

Navigating a fragile capability signifies a profound tension between professional agency and existential exposure, where the boundaries of competence are brought to light. This is characterised by being vulnerable under the burden of responsibility, a paradoxical state of powerlessness within a position of power, where standardised protocols fail to reach the depth of a complex existential crisis. Facing potentially fatal consequences necessitates pivotal decisions in a situation that defies control, leaving a sense of frustration when suffering cannot be resolved through technical means alone. In this state of exposure, a transition from doing to being becomes necessary, leading toward bridging isolation through a shared humanity. By stepping beyond the uniform to seek a human-to-human connection, a reciprocal space emerges where the desire to provide support is inseparable from being emotionally moved. Here, professional capability is reshaped through the embodying of solicitude and a shared understanding of the crisis. Through listening and allowing one’s own narrative to be influenced by the encounter, the meaning of the encounter shifts toward a presence where the fragility of the situation is no longer a failure of competence but a prerequisite for a genuine encounter

#### Being vulnerable under the burden of responsibility

Encountering a patient in a suicidal process means being vulnerable under the burden of responsibility. It implies being powerless, paradoxically in a position of power, and finding oneself in the void where standardised protocols fail to reach the depth of a complex existential crisis. Facing potentially fatal consequences necessitates the pressure of making pivotal decisions as part of the burden of responsibility. Ultimately, it means facing the frustration of being unable to resolve the suffering and standing exposed in a situation that cannot be controlled.

##### Being professionally and existentially powerless.

To encounter a patient in a suicidal process means balancing a paradox of being in a position of authority while being inherently powerless. It is to find oneself where protocols fail to reach the patient’s inner suffering. Being unable to reach or connect with the person in crisis involves an experience of futility. It is a state where the absence of tangible actions results in frustration. The situation necessitates a reliance on one’s personal self, as conventional protocols offer no support. This means being exposed and without tools when a barrier obstructs the normal process of care. Being professionally capable is strained by the absence of standardised procedures to resolve a complex existential crisis rather than physical trauma. The encounter reveals that professional mastery is insufficient when faced with the absolute vulnerability of another human being.

*‘As a fellow human being, you feel quite sad that she’s ended up there (…) and to feel so helpless yet still be the only one who can help. I feel, I understand that I don’t have the power or the knowledge to just press a button and get her out of this’.* (Participant 15)

The encounter entails a solitary responsibility, where the patient’s entire life is experienced as resting on one’s shoulders. It means searching for guidance in training and protocols but meeting an echoing silence, leaving the clinicians in an existential vacuum.

*‘When you’re at the back of the vehicle—no then it’s only me and him and that’s when you feel even more alone. Then somehow, it’s up to me in the conversation, and it might be a scary feeling too or a little hard’.* (Participant 9)

Furthermore, the encounter means being in a position of professional power yet being existentially disempowered when the system fails the patient in crisis. Efforts are experienced as being in vain when the subsequent transition to psychiatric care is met with hopelessness and a lack of confidence in the receiving personnel.

*‘When she sees which staff members are working at the psychiatric emergency department, she’s just like, ‘Oh is it him? Well, I’m certainly not going to get any help today.’ And then, you almost get a lump in your throat. I’ve tried and tried and tried to get her to come with me, and then that’s just the response you get’.* (Participant 7)

This reflects a state where the capability to help is undermined by the failure of the system in its entirety. Ultimately, this powerlessness signifies that capability is not an isolated professional attribute but is fundamentally dependent on a functioning relational and systemic context, where a failure in the chain of care translates into an individual existential burden.

##### Balancing professional composure against internal fragility.

To encounter a patient in a suicidal process means assuming a position of protective authority, balancing the immediate need for physical safety with the internal demand for professional composure. It implies a state of dual vigilance, being observant of both the patient and the environment to identify possible threats, while simultaneously composing one’s own emotions and physical reactions.

*‘I definitely get a higher pulse, which I’ve noticed that I get when I don’t know what to do. I’m really calm, even in emergency situations, if I know what to do. But when I don’t know what to do, that’s when I get nervous’.* (Participant 16)

Encountering the patient in a suicidal process means treading carefully, striving to maintain a calm atmosphere to avoid upsetting the patient before specialised care can be established. Silence is accepted and reflects strategic navigation weighed against the risk of initiating a conversation that may exceed one’s communicative capacity. This endeavour involves fostering trust in the healthcare system and ensuring the patient’s transition into psychiatric expertise. Given the potentially fatal consequences, this responsibility entails the pressure of making pivotal decisions. In such critical situations, where so much is at stake, it means being prepared for the worst while resolutely striving for the best possible outcome.

*‘Nobody else would get out on the roof, but I felt pretty safe and went out on the roof. It felt precarious because it’s not somebody sitting in their living room expressing sadness. But he did it in a situation where he could have only taken one step and killed himself. So, it felt extra crucial and sensitive that you don’t do anything wrong’.* (Participant 12)

Even when individual autonomy is acknowledged, the encounter occasionally necessitates overstepping the patient’s agency in decision-making. For instance, by securing transport to the hospital, despite an expressed refusal of care. It involves ensuring control through actions, at times even physically interrupting ongoing suicidal action. The encounter entails balancing the thin line between providing help and committing abuse. Encountering a patient in a suicidal process challenges the struggle for control and reveals the limitation of facing a patient whose internal will to live cannot be externally controlled.

##### Being indecisive in the void of protocols.

To encounter a patient in a suicidal process means to progress in the void of conventional protocols and the feedback mechanisms normally relied upon. The encounter entails a loss of professional certainty, as it requires a different set of skills, mainly based on communicative and interpersonal competence. Uncertainty arises regarding what to say or do, signifying that the helpfulness of an intervention remains unknown.

*‘I can feel inadequate because if I come to you and you’ve broken your leg, I know exactly what to do. If you say your chest hurts and you’re having a heart attack, I know exactly (!) what to do. But your soul is broken… I feel like well, what should I say? What should I do? I can’t heal you!’* (Participant 2)

A patient who does not engage in conversation means an impediment to the natural flow of an assignment. Uncomfortable silence in the encounter necessitates a scrutiny of the situation to determine alternative actions. When nothing appears to work and previous experiences fail to untangle a blocked situation, the encounter is characterised by a standstill.

*‘I tried in the way that I’ve previously been able to reach her, I’ve even been able to joke with her. But this time it didn’t work and then I become a little… I get a mental block; it gets really hard and then I don’t really know’…*(Participant 3)

Indecisiveness occurs in the void left by inadequate protocols. Having no universal manual for genuine human encounter means hesitating cautiously, as both words and silence become heavy with consequence. This indecisiveness reflects a double silence, where the withdrawal of the patient and the silence of the guidelines leave the clinician without a clear direction, turning the encounter into a wordless struggle for connection.

#### Bridging isolation through a shared humanity

Bridging isolation through a shared humanity means being personal beyond the uniform, seeking connection in a human-to-human relationship where interpersonal competence allows for a shared understanding of the crisis. It implies a transition from doing to being and means embodying solicitude. To bridge isolation through shared humanity means entering a reciprocal space where the desire to support is inseparable from being emotionally moved. It means remaining present through listening and allowing one’s own narrative to be reshaped by the encounter with the patient

##### Being let in.

Encountering a patient in a suicidal process means seeking connection and establishing a relationship rooted in cooperation. The encounter involves meeting the patient in a human-to-human relationship, being personal beyond the professional uniform. This necessitates complementing standardised training with interpersonal competence. Through conversation, it becomes possible to connect with the patient and reach a shared understanding of the situation. Having established this connection implies a sense of ease where the specific choice of words becomes less of a concern. At the same time, attuning to a calm and comfortable dialogue can be difficult if the preceding moments are characterised by intensity and stress.

*‘It felt really good [to connect with the patient], but at the same time I’d been through this enormously pressured situation, just a few seconds earlier. So, my pulse was up, and I was all wound up and felt stressed! At the same time, you’re supposed to appear as professional as possible and just switch—okay, now we’re going to talk nice and easy’.* (Participant 1)

The encounter becomes meaningful when a glimmer of hope arises in the patient, and the horizon of potentially saving a life signifies an achievement comparable to treating a life-threatening somatic condition. Confirmation and pride occur when a patient shows trust and discloses suicidal ideation. A personal approach allows clinicians to enter the patient’s world, which bridges professional distance and provides the opportunity to offer genuine support.

##### Embodying solicitude through presence.

Encountering a patient in a suicidal process means embodying solicitude through presence, signifying a striving to offer a sense of togetherness within the darkness of the crisis. Being present means accepting the situation without the ability to alter the past or the knowledge of what the future holds for the patient. Although the time with the patient is limited, it can hold a qualitative depth. Understanding that the encounter takes place during a moment of crisis for the patient means believing that better times can come. The encounter means caring through time, allowing the interaction to take the necessary time to support the patient through the crisis.

*‘If you think about how it was two or three hours of my life that saved her life—If I think about it that way, it’s really a mere nothing of my life. Then I’m happy to be completely wrung out afterwards’.* (Participant 18)

The awareness that people can endure a crisis sustains the effort for the patient in the current situation. Focusing on the immediate situation implies a necessary distanciation from hopelessness, especially when encountering a patient repeatedly. Focusing on the present also makes the situation manageable, as it can be overwhelming to encompass both the history behind the crisis and the journey that awaits the patient. Ultimately, being present means a commitment to not abandon the patient, signifying the capacity to remain in the encounter amidst absolute vulnerability.

##### Becoming part of each other’s narratives.

To encounter a patient in a suicidal process means entering the middle of a narrative without knowing its origin or direction. When a patient engages in conversation, a possibility emerges to understand the situation and obtain explanations as to why the patient is in a suicidal process.

*‘When she tells me what she’s been through, how things have been and how life and society have treated her, then I get a much deeper understanding of why she’s where she’s at. I mean, I can only feel sorry for her, but I can understand why she’s ended up here’.* (Participant 15)

The encounter implies allowing oneself to be emotionally affected when witnessing a distress so profound that suicide is seen as the only option. Adhering to the narrative requires the strength to remain present while listening to the darkness of the patient’s experience. Within this listening, the possibility emerges to discern fragments of hope. As there is no formal follow-up, a lingering remains, reflecting thoughts about the patient’s fate and outcome. This entails reflecting on whether the efforts made were helpful and if the encounter truly left an impact. Occasionally, this uncertainty is resolved by encountering a former patient and seeing that their narrative has turned toward life.

*‘Today I run into her working in a supermarket, and when she says ‘have a nice day’, I don’t think she knows who I am, but it makes me feel so warm inside every time I see her. And she seems to have a purpose—she has a job; she seems to like it there’.* (Participant 7)

Listening to the narrative also provides a way of discovering suicidality in a patient whose initial cause of contact is somatic. It means adhering to signs of weariness, loneliness or hopelessness to understand their meaning within the patient’s narrative. Encountering a patient in a suicidal process means entering a shared narrative space at a point of crisis. This implies a reciprocal movement where the aim to impact the patient’s journey is inseparable from being emotionally moved and having one’s own narrative reshaped in the encounter with the patient.

### Comprehensive understanding

The main theme, *navigating a fragile capability*, can be understood as a profound tension within the ontology of *the capable human being* (Ricœur, 2005, 1992). While the professional identity of ambulance clinicians is traditionally anchored in *I can*—the ability to speak, act, narrate, and be accountable, the encounter with a patient in a suicidal process imposes a transition from a state of agency to a state of passivity and suffering. This movement renders the professional identity fragile and reveals that capability is not a static attribute, but a construct fundamentally challenged by a double silence, characterised by the patient’s withdrawal and the experienced silence of guidelines. In the absence of protocols and feedback, a crisis of attestation emerges, signifying that the clinician’s own sense of agency is inherently validated through the patient. If the patient refuses to be helped or declines to participate in a shared narrative, the capacity to act is fractured. This dialectic reflects that professional identity is inseparable from the response of the patient. However, it is in this *vulnerability under the burden of responsibility* that a deeper, more ethical form of capability is born. This transition marks a shift from technical competence towards phronesis, or practical wisdom. By bridging isolation through a shared humanity, the clinicians acknowledge the patient not as a medical object to be resolved, but as a suffering subject whose narrative is momentarily blocked. Being capable is thus no longer defined by maintaining control or fixing a problem, but by the courage to remain present in the darkness. This reflects an embodiment of solicitude, where the clinician’s capability is reshaped into the ethical capacity to endure vulnerability alongside the patient. In this reciprocal space, the encounter reaches its ultimate meaning, in what Ricœur (1992) defines as the ethical aim: “the good life, with and for others, in just institutions”.

## Discussion

The findings demonstrate that encountering patients in a suicidal process challenges the traditional prehospital emphasis on technical agency, revealing a profound tension between professional control and existential vulnerability. Rather than relying solely on standardised procedures, clinicians are forced to navigate a space where ethical presence and relational competence become essential when protocols reach their limit

### Professional identity and technical agency

The experiences of fragility and passivity described by clinicians in this study highlight how the double silence of the encounter threatens the traditional foundations of prehospital care. While ambulance clinicians often focus on technical medical procedures to achieve emotional detachment and handle acute stress during critical incidents (Avraham et al., 2014), this professional construct is rendered vulnerable when facing the vacuum between the patient’s withdrawal and the silence of guidelines. Structural voids of this nature effectively remove conventional feedback mechanisms, disrupting the natural flow of the assignment. Such a disruption of professional agency highlights how existential passivity directly undermines the clinicians’ fundamental need to actively manage the scene and control the environment to facilitate medical interventions (Campeau, 2008).

Navigating these professional boundaries underscores a fundamental discrepancy between the healthcare system’s heavy focus on safety and the actual, chaotic nature of an existential crisis. A suicidal crisis involves a relational collapse where everyday ways of understanding the world and oneself cease to function (Roennfeldt et al., 2024). In such a state, patients may perceive clinical risk management and safety protocols as irrelevant or even harmful, as these measures fail to address the core loss of meaning. While clinicians feel restricted by the silence of protocols, this void represents the very space where the patient’s need for relational contact can be met, and the possibility of a restored narrative can unfold. However, when conventional clinical indicators and patient responsiveness are absent, the clinicians’ fundamental trust in their own ability is shaken, leading to what Ricœur (1992) defines as a crisis of attestation. The transition reveals that the clinicians’ sense of agency is deeply dependent on the patient’s reciprocity and is inherently validated through the patient. When this reciprocity is missing, technical proficiency alone proves insufficient to address the patient’s internal state, highlighting a struggle to find meaning in presence alone.

For the clinicians, a complex shift reflects the tension between what Fredriksson (1999) defines as contact versus connection. While task-oriented contact allows the clinician to be present through routine actions, the encounters described in this study reveal a demand for a transition from *being there* to *being with* the patient in active listening and genuine presence. Navigating the difficulty of this transition is underscored by ambulance clinicians’ common use of emotional distancing as a strategy to manage professional stress (Almeida et al., 2023), making the choice to remain present an even more significant ethical stance. Furthermore, the unique role of the ambulance service acts as an invisible witness to the community’s suffering, capturing calls of despair that never reach the hospital system (Bellamy et al., 2025).

### Relational presence and ethical competence

Experiencing this resulting state of passivity can be perceived as a failure to act, especially if the clinicians’ competence is primarily linked to the practical and task-oriented dimensions of care. Consequently, this perceived lack of competence and the subsequent sense of indecisiveness identified in the present study stand in contrast to the results of Önnheim et al. (2022), where ambulance clinicians reported their overall competence in psychiatric encounters to be high. It is plausible that self-assessments of competence are linked to the practical and task-oriented dimensions of care, rather than the complex, relational demands of remaining present with a patient in a suicidal process. Even though ambulance clinicians play a pivotal role in suicide prevention through assessment and identification (Doan et al., 2024), a rigid task-oriented focus may obscure the existential demands of the encounter.

Rooted in a broader educational focus, the preference for technical agency may develop early. As Todorova et al. (2021) note, curricula within ambulance education traditionally emphasise somatic diseases, leading clinicians to concentrate on medical disorders at the expense of mental health care. Such an educational bias is reinforced by a professional culture that often perceives mental health presentations as being outside the core business of emergency care (Brewster et al., 2022). Furthermore, organisational influences that prioritise safety strategies and professional distance can inadvertently lead to the othering of patients, where the person is viewed more as a risk to be managed than a person to be met (Björklund et al., 2025). To counter this othering, the clinician can be understood as functioning as a narrative guardian within the crisis, a protective presence that safeguards the space of a shared humanity without rewriting the patient’s story or assuming a specific trajectory. Moving beyond mere risk assessment, the encounter becomes a space where the patient’s narrative identity is impacted by representatives of society (Hammarbäck et al., 2024). Within this perspective, an encounter grounded in shared humanity rather than clinical distance opens the possibility for patients to shift from feeling like a burden toward regaining human value. Recognising the contrast between the clinicians’ perceived powerlessness and the patient’s need for contact suggests that relational presence may function as a vital anchor. Supporting a person in this acute phase can thus be understood as moving beyond strictly preserving life to safeguarding a space where a coherent life story might eventually be rebuilt. Failing this transition carries risks underscored by Macdonald et al. (2020), who note that negative experiences of emergency care can increase the risk of future suicidal actions. Wihlborg et al. (2017) argue that professional development is strongly dependent on the ability to reflect on practice. Since clinicians’ perceptions of their competence are closely linked to patient outcomes, the experienced sense of powerlessness identifies an urgent need for reflective spaces, where the existential dimensions of the encounter can be integrated into the professional identity.

Ultimately, in light of Ricœur’s philosophy (1992), the uncertainty experienced when protocols fall silent can be understood as an expression of moral sensitivity rather than a lack of competence. Recognising vulnerability as an inherent dimension of practice serves as the foundation for true ethical judgement, highlighting that relational presence is an integral component of professional capability.

### Organisational context and international transferability

Existential powerlessness and clinical indecisiveness emerge as central barriers when ambulance clinicians encounter a suicidal process in the silence of protocols. While ambulance service managers emphasise the importance of holistic care (Holmberg et al., 2017), the present findings suggest that clinicians lack organisational support to handle the existential powerlessness they encounter when a patient’s narrative is momentarily blocked. Analysing this lack of support requires examining the specific organisational and legal context of the study. Bergman et al. (2025) highlight a major objective deficit in this domain, demonstrating that four out of ten psychiatric prehospital presentations completely lack a priority assessment due to standard tools focusing almost exclusively on physical, somatic signs. When standard triage systems fail to validate existential distress, the clinicians are left to carry the weight of an unguided clinical decision.

Overriding a patient’s refusal of care to safeguard their life introduces a fundamental conflict with self-determination. Ambulance clinicians frequently experience moral distress when forced to balance autonomy against a patient’s perceived best interest (Bremer & Holmberg, 2020), particularly when a breakdown in communication blocks access to the patient’s narrative. Evaluating these systemic conditions carries important implications for how the findings can be transferred to ambulance systems internationally. As a comparative policy analysis by Mordaunt et al. (2026) demonstrates, legal mandates regarding involuntary psychiatric interventions vary significantly across jurisdictions. While some international models grant ambulance clinicians explicit clinical autonomy to independently detain at-risk individuals, the Swedish framework lacks specific clinical protocols for restraint, forcing the clinicians to rely on general citizen-level legislation regarding the principle of necessity. Consequently, operating under non-clinical legislation heavily complicates immediate risk management, contributing to the experiences of vulnerability and role confusion identified in this study.

Choosing to bridge isolation through a shared humanity requires clinicians to actively transition from a task-oriented focus toward an authentic relational presence. Parker et al. (2022) note that caring interactions require emergency clinicians to balance complex clinical *doing* with humanistic *being*. Stepping beyond uniformed authority to establish an emotional presence effectively humanises the encounter, transforming the prehospital workspace from a logistical transport utility into a safe haven where a patient’s narrative identity (Ricœur, 1992) can be preserved amidst an existential collapse. Prioritising the patient’s perspective to reduce existential suffering is exceptionally critical for vulnerable populations in a suicidal process, where cold or dismissive staff encounters reinforce intense shame and deter individuals from seeking future emergency or psychiatric help.

## Methodological considerations

Regarding the choice of method, the phenomenological hermeneutical approach (Lindseth & Norberg, 2022) was selected to reach the ontological level of the phenomenon. While interpretative phenomenological analysis (Smith et al., 2009) was considered, it was found to remain closer to individual experiences rather than the phenomenon itself. The current approach, inspired by the philosophy of Ricœur (1991), allows for a more robust rigour through the hermeneutic arch, capturing both understanding and explanation. Although the Critical Incident Technique (CIT) (Flanagan, 1954) could have addressed the critical nature of these assignments, it was deemed insufficient for this study. While the CIT typically focuses on identifying functional behaviours or specific outcomes, a phenomenological hermeneutical approach made it possible to elucidate the deeper existential meanings and fragility inherent in the ambulance clinicians’ capability.

A potential limitation is that the shared narratives often originated from either the most recent assignments or extraordinary experiences. While this could be perceived as a constraint on variation, it is consistent with the idea that significant encounters, those that carry weight and meaning, are more readily narrated. These narratives provided a rich entry point into the lived experience of the phenomenon. Although the interviews often began with a specific incident, the narrative process enabled participants to reflect on and contrast these with more ordinary assignments, thereby contributing to the comprehensive richness of meaning. In relation to this narrative depth, the sample size of 18 participants was considered sufficient, as the quality of the data provided the necessary density to illuminate the complexity of the phenomenon.

A further methodological consideration is the potential risk of selection bias, as clinicians who volunteered to participate in this study and discuss these emotionally demanding encounters may possess different characteristics or coping strategies than those who chose not to participate. Additionally, the collected data rely on the participants’ memories, meaning that the narratives could have been influenced by retrospective recall. The moral distress experienced during the encounters, combined with the effects of later professional reflection over time, might also have reshaped how these experiences were recalled and narrated during the interviews.

In line with the phenomenological hermeneutical tradition (Lindseth & Norberg, 2004, 2022), the rigour of this study is evaluated through the cogency and plausibility of the interpretation rather than traditional criteria for reliability (Ricœur, 1976). This involved balancing the tension between a hermeneutics of faith, characterised by an openness to the participants’ narrated meanings, and a hermeneutics of suspicion, where the structural analysis serves to challenge and deconstruct initial understandings. To ensure a truthful fiction, a coherent and re-contextualised interpretation that captures the essential meaning of the lived experience, the researchers engaged in a continuous movement between the parts and the whole in the hermeneutic arch. This process allowed for a critical distance where the authors’ combined preunderstanding was used as a tool for sensitivity, while the dialectic between trust and suspicion ensured that the final interpretation remained grounded yet critically reflective. The final interpretation was reached through continuous dialogue within the research team, where emerging themes were compared and refined until a consensus on the most plausible understanding was achieved. The trustworthiness of the findings resides in the transparency of this interpretive process, offering a meaningful and coherent understanding of the phenomenon. Furthermore, the transferability of the findings is supported by thick descriptions of the participants’ narrated experiences, allowing readers to assess the applicability of the meanings to other emergency care contexts.

Future research directions could benefit from evaluating specific suicide-related communication training programs within prehospital care. It would also be valuable to conduct similar studies in different ambulance systems internationally to explore how various organisational and legal frameworks influence the encounter. Furthermore, future studies comparing clinicians’ perspectives with the experiences of the patients themselves would provide a more comprehensive understanding of the relational dynamics. Finally, investigating the implementation of structured post-event reflection and its specific effects on moral distress and professional confidence among ambulance clinicians, represents a crucial area for further enquiry.

## Conclusion

Encountering patients in a suicidal process reveals that professional capability is not a static attribute, but a fragile construct that is fundamentally dependent on reciprocity. When facing the double silence of patient withdrawal and the vacuum of guidelines, the meaning of care shifts from technical agency toward an ethical presence. Capability in these encounters is defined not by resolving a medical problem alone, but by the courage to remain present in shared humanity, where vulnerability emerges as an inherent dimension of practical wisdom. Consequently, the clinicians’ experiences of indecisiveness and powerlessness can be understood as expressions of moral sensitivity rather than professional inadequacy. By validating *being with* as a clinically meaningful component of care, the encounter can move beyond procedural care toward a meaningful ethical aim. Ultimately, the clinician functions as a narrative guardian within the existential crisis, offering a protective presence that safeguards the space of a shared humanity without assuming a specific patient outcome or future trajectory.

## Implications

- Professional standards and ambulance education curricula should strive to move beyond technical management and risk assessment to integrate relational competence and existential care as core elements of prehospital practice. This includes introducing specific training modules focusing on crisis communication and the existential dimensions of psychiatric care to counter the traditional somatic bias in education.
- Ambulance organisations should provide structured spaces for ethical reflection, which may help mitigate moral distress, acknowledging that the ability to navigate existential powerlessness is a sophisticated clinical skill that requires ongoing support. To remain operationally feasible, these existential and ethical dimensions should be integrated into existing routines, such as case reviews and scenario training, embedding mental health dilemmas alongside somatic care.
- Validating presence as a vital and legitimate action can help clinicians regain trust in their own capability and strengthen their professional attestation when technical protocols are insufficient. Leadership needs to actively value and encourage this competence, fostering an organisational culture where clinicians are supported to be fully present and listening during the time they have with the patient, regardless of whether the encounter is psychiatric, somatic, or a complex combination of both.
- The professional culture and leadership within ambulance care should be challenged to shift from an emphasis on technical action toward valuing the quality of being. Prioritising this presence could support clinicians in feeling professionally secure when maintaining an ethical presence, ensuring that the absence of technical interventions is not perceived as a lack of competence or a professional failure.

## Supplementary Material

Supplementary Material SRQR_Checklist.pdf

## Data Availability

The data that support the findings of this study are not publicly available due to their containing information that could compromise the privacy of research participants. Due to the sensitive nature of the interviews and the risk of re-identification, the data are not shared.

## References

[cit0001] Ahmedani, B. K. , Westphal, J. , Autio, K. , Elsiss, F. , Peterson, E. L. , Beck, A. , Waitzfelder, B. E. , Rossom, R. C. , Owen-Smith, A. A. , Lynch, F. , Lu, C. Y. , Frank, C. , Prabhakar, D. , Braciszewski, J. M. , Miller-Matero, L. R. , Yeh, H. H. , Hu, Y. , Doshi, R. , Waring, S. C. , & Simon, G. E. (2019). Variation in patterns of health care before suicide: A population case-control study. *Preventive Medicine* , *127* . 105796. 10.1016/j.ypmed.2019.105796 31400374 PMC6744956

[cit0002] Almeida, M. , Lobão, C. , Coelho, A. , & Parola, V. (2023). Emotional management strategies in prehospital nurses: A scoping review. *Nursing Reports* , *13* (4), 1524–1538. 10.3390/nursrep13040128 37987407 PMC10661275

[cit0003] Avraham, N. , Goldblatt, H. , & Yafe, E. (2014). Paramedics’ experiences and coping strategies when encountering critical incidents. *Qualitative Health Research* , *24* (2), 194–208. 10.1177/1049732313519867 24495988

[cit0004] Bayliss, L. T. , Lamont-Mills, A. , & du Plessis, C. (2025). I will die by my own Hand”: Understanding the development of suicide capability in the narratives of individuals who have attempted suicide. *Qualitative Health Research* , *35* (6), 589–600. 10.1177/10497323241235861 38914024 PMC12041613

[cit0005] Bellamy, V. , Wilcock, H. , Wilson, C. , & Crabtree, R. (2025). Patterns and characteristics of ‘calls of despair’: A service evaluation using yorkshire ambulance service data. *British Paramedic Journal* , *10* (2), 40–48. 10.29045/14784726.2025.9.10.2.40 40904794 PMC12404226

[cit0006] Bergman, N. , Jarling, A. , Norberg, G. B. , Alenljung, B. , & Andersson, M. H. (2025). Description of patients presenting with mental illness in emergency medical services: A retrospective observational study. *Scandinavian Journal of Trauma, Resuscitation and Emergency Medicine* , *33* (1), 139. 10.1186/s13049-025-01453-9 40804695 PMC12344978

[cit0007] Björklund, S. , Hagell, P. , Holmberg, M. , & Lilja Hagell, P. (2025). Influences on ambulance staff’s understandings and safeguarding of ethical values. *Nursing Ethics* , *32* (7), 2431–2444. 10.1177/09697330251344170 40420557 PMC12550201

[cit0008] Bouveng, O. , Bengtsson, F. A. , & Carlborg, A. (2017). First-year follow-up of the psychiatric emergency response team (PAM) in Stockholm county, Sweden: A descriptive study. *International Journal of Mental Health* , *46* (2), 65–73. 10.1080/00207411.2016.1264040

[cit0009] Bremer, A. , & Holmberg, M. (2020). Ethical conflicts in patient relationships: Experiences of ambulance nursing students. *Nursing Ethics* , *27* (4), 946–959. 10.1177/0969733020911077 32253975 PMC7323741

[cit0010] Brewster, L. , Bear, R. , & Maria, S. (2022). Overcoming stigma of mental illness in paramedicine: A model for future practice. *Australasian Journal of Paramedicine* , *19* , 1–11. 10.33151/ajp.19.1023

[cit0011] Brinkmann, S. , & Kvale, S. (2015). *InterViews: learning the craft of qualitative research interviewing* . Sage Publications.

[cit0012] Brottsbalk [Swedish Penal Code] (SFS 1962:700). Riksdagen. https://www.riksdagen.se/sv/dokument-lagar/dokument/svensk-forfattningssamling/brottsbalk-1962700_sfs-1962-700

[cit0013] Campeau, A. G. (2008). The space-control theory of paramedic scene-management. *Symbolic Interaction* , *31* (3), 285–302. 10.1525/si.2008.31.3.285

[cit0014] Dickson-Swift, V. , James, E. L. , Kippen, S. , & Liamputtong, P. (2007). Doing sensitive research: What challenges do qualitative researchers face?. *Qualitative Research* , *7* (3), 327–353. 10.1177/1468794107078515

[cit0015] Doan, T. N. , Rashford, S. , Sims, L. , Wilson, K. , Garner, S. , & Bosley, E. (2024). Suicide-related out-of-hospital cardiac arrests in Queensland, Australia: Temporal trends of characteristics and outcomes over 14 years. *Prehospital Emergency Care* , *28* (3), 431–437. 10.1080/10903127.2023.2230595 37364032

[cit0016] Flanagan, J. C. (1954). The critical incident technique. *Psychological Bulletin* , *51* (4), 327–358. 10.1037/h0061470 13177800

[cit0017] Fredriksson, L. (1999). Modes of relating in a caring conversation: A research synthesis on presence, touch and listening. *Journal of Advanced Nursing* , *30* (5), 1167–1176. 10.1046/j.1365-2648.1999.01192.x 10564416

[cit0018] Hälso- och sjukvårdslag [Health and Medical Services Act] (SFS 2017:30). Riksdagen. https://www.riksdagen.se/sv/dokument-lagar/dokument/svensk-forfattningssamling/halso--och-sjukvardslag-201730_sfs-2017-30

[cit0019] Hammarbäck, S. , Holmberg, M. , Wiklund Gustin, L. , & Bremer, A. (2023). Ambulance clinicians’ responsibility when encountering patients in a suicidal process. *Nursing Ethics* , *30* (6), 857–870. 10.1177/09697330221149102 37026403 PMC10637079

[cit0020] Hammarbäck, S. , Wiklund Gustin, L. , Bremer, A. , & Holmberg, M. (2024). Navigating oneself through the eyes of the other – meanings of encountering ambulance clinicians while being in a suicidal process. *International Journal of Qualitative Studies on Health and Well-being* , *19* (1), 2374751. 10.1080/17482631.2024.2374751 38954758 PMC11221472

[cit0021] Holmberg, M. , Fagerberg, I. , & Wahlberg, A. C. (2017). The knowledge desired by emergency medical service managers of their ambulance clinicians – A modified delphi study. *International Emergency Nursing* , *34* , 23–28. 10.1016/j.ienj.2017.03.007 28545930

[cit0022] Husserl, E. (2014). *Ideas for a pure phenomenology and phenomenological philosophy. First book, General introduction to pure phenomenology* . Hackett Publishing Company.

[cit0023] Knipe, D. , Padmanathan, P. , Newton-Howes, G. , Chan, L. F. , & Kapur, N. (2022). Suicide and self-harm. *The Lancet* , *399* (10338), 1903–1916. 10.1016/S0140-6736(22)00173-8 35512727

[cit0024] Lag om psykiatrisk tvångsvård [Compulsory Psychiatric Care Act] (SFS 1991:1128). Riksdagen. https://www.riksdagen.se/sv/dokument-lagar/dokument/svensk-forfattningssamling/lag-19911128-om-psykiatrisk-tvangsvard_sfs-1991-1128

[cit0025] Larsen, J. L. S. , Hawton, K. , Silverman, M. , Nordentoft, M. , & Erlangsen, A. (2025). Ambivalence and suicidal behavior at railway stations: A scoping review. *Archives of Suicide Research* , Epub ahead of print, 1–20. 10.1080/13811118.2025.2573065 41159631

[cit0026] Lindseth, A. , & Norberg, A. (2004). A phenomenological hermeneutical method for researching lived experience. *Scandinavian Journal of Caring Sciences* , *18* (2), 145–153. 10.1111/j.1471-6712.2004.00258.x 15147477

[cit0027] Lindseth, A. , & Norberg, A. (2022). Elucidating the meaning of life world phenomena. A phenomenological hermeneutical method for researching lived experience. *Scandinavian Journal of Caring Sciences* , *36* (3), 883–890. 10.1111/scs.13039 34687247

[cit0028] Lindström, V. , Sturesson, L. , & Carlborg, A. (2020). Patients’ experiences of the caring encounter with the psychiatric emergency response team in the emergency medical service-A qualitative interview study. *Health Expectations* , *23* (2), 442–449. 10.1111/hex.13024 31967699 PMC7104631

[cit0029] Macdonald, S. , Sampson, C. , Turley, R. , Biddle, L. , Ring, N. , Begley, R. , & Evans, R. (2020). Patients’ experiences of emergency hospital care following self-harm: Systematic review and thematic synthesis of qualitative research. *Qualitative Health Research* , *30* (3), 471–485. 10.1177/1049732319886566 31933427

[cit0030] Malterud, K. , Siersma, V. D. , & Guassora, A. D. (2016). Sample size in qualitative interview studies: Guided by information power. *Qualitative Health Research* , *26* (13), 1753–1760. 10.1177/1049732315617444 26613970

[cit0031] Marcinkevičiūtė, M. , Vilutytė, L. , & Gailienė, D. (2024). Experience of pre-suicidal suffering: Insights from suicide attempt survivors. *International Journal of Qualitative Studies on Health and Well-being* , *19* (1), 2370894. 10.1080/17482631.2024.2370894 38913782 PMC11198122

[cit0032] Mordaunt, D. A. , O’Byrne, D. , & Jones, N. (2026). Paramedic powers in mental health crises: A comparative legal analysis. *Australian & New Zealand Journal of Psychiatry* , *60* (2), 184–190. 10.1177/00048674251395412 41355235 PMC12831802

[cit0033] Nilsson, C. , Bremer, A. , Blomberg, K. , & Svantesson, M. (2017). Responsibility and compassion in prehospital support to survivors of suicide victim - professionals’ experiences. *International Emergency Nursing* , *35* , 37–42. 10.1016/j.ienj.2017.06.004 28687433

[cit0034] O’Brien, B. C. , Harris, I. B. , Beckman, T. J. , Reed, D. A. , & Cook, D. A. (2014). Standards for reporting qualitative research: A synthesis of recommendations. *Academic Medicine* , *89* (9), 1245–1251. 10.1097/ACM.0000000000000388 24979285

[cit0035] Önnheim, S. , Johansson, A. , Ivarsson, B. , & Hagström, C. (2022). Self-perceived competence of ambulance nurses in the care of patients with mental illness: A questionnaire survey. *Nursing Reports* , *12* (1), 226–234. 10.3390/nursrep12010023 35324569 PMC8954289

[cit0036] Parker, L. , Prior, S. J. , Van Dam, P. J. , & Edwards, D. G. (2022). Altruism in paramedicine: A scoping review. Healthcare, *10* (9), 1731. 10.3390/healthcare10091731 36141343 PMC9498595

[cit0037] Rees, N. , Rapport, F. , & Snooks, H. (2015). Perceptions of paramedics and emergency staff about the care they provide to people who self-harm: Constructivist metasynthesis of the qualitative literature. *Journal of Psychosomatic Research* , *78* (6), 529–535. 10.1016/j.jpsychores.2015.03.007 25819635

[cit0038] Rees, N. , Porter, A. , Rapport, F. , Hughes, S. , & John, A. (2018). Paramedics’ perceptions of the care they provide to people who self-harm: A qualitative study using evolved grounded theory methodology. PLoS One, *13* (10), e0205813. 10.1371/journal.pone.0205813 30332480 PMC6192640

[cit0039] Ricœur, P. (1976). *Interpretation theory: discourse and the surplus of meaning* . Texas Christian Univ. Press.

[cit0040] Ricœur, P. (1991). *From text to action: essays in hermeneutics II* . Athlone.

[cit0041] Ricœur, P. (1992). *Oneself as another* . Univ. of Chicago Press.

[cit0042] Ricœur, P. (2005). *The course of recognition* . Harvard University Press.

[cit0043] Roberts, L. , Masters, S. , & Henderson, J. (2025). Reviewing Australian paramedic clinical practice guidelines for persons experiencing a mental health crisis. *Australasian Emergency Care* , 29(2), 125–137. 10.1016/j.auec.2025.09.007 41093688

[cit0044] Roennfeldt, H. , Hill, N. , Byrne, L. , & Hamilton, B. (2024). The anatomy of crisis. *International Journal of Qualitative Studies on Health and Well-being* , *19* (1). 2416580. 10.1080/17482631.2024.2416580 39417632 PMC11488168

[cit0045] Romeu, D. , Guthrie, E. , & Mason, S. M. (2023). Understanding prehospital care for self-harm: Views and experiences of yorkshire ambulance service clinicians. *Emergency Medicine Journal* , *40* (7), 482–483. 10.1136/emermed-2022-212983 36792341

[cit0046] Shamsaei, F. , Yaghmaei, S. , & Haghighi, M. (2020). Exploring the lived experiences of the suicide attempt survivors: A phenomenological approach. *International Journal of Qualitative Studies on Health and Well-being* , *15* (1), 1745478. 10.1080/17482631.2020.1745478 32223374 PMC7172699

[cit0047] Shin, H. D. , Price, S. , & Aston, M. (2021). A poststructural analysis: Current practices for suicide prevention by nurses in the emergency department and areas of improvement. *Journal of Clinical Nursing* , *30* (1-2), 287–297. 10.1111/jocn.15502 32956549

[cit0048] Sjölin, H. , Lindström, V. , Hult, H. , Ringsted, C. , & Kurland, L. (2019). Common core content in education for nurses in ambulance care in Sweden, Finland and Belgium. *Nurse Education in Practice* , *38* , 34–39. 10.1016/j.nepr.2019.05.017 31176241

[cit0049] Smith, J. A. , Flowers, P. , & Larkin, M. (2009). *Interpretative phenomenological analysis: theory, method and research* . Sage.

[cit0050] Todorova, L. , Johansson, A. , & Ivarsson, B. (2021). Perceptions of ambulance nurses on their knowledge and competence when assessing psychiatric mental illness. *Nursing Open* , *8* (2), 946–956. 10.1002/nop2.703 33570281 PMC7877124

[cit0051] Turecki, G. , & Brent, D. A. (2016). Suicide and suicidal behaviour. *The Lancet* , *387* (10024), 1227–1239. 10.1016/s0140-6736(15)00234-2 PMC5319859 26385066

[cit0052] Wasserman, D. (2016). The suicidal process. In I. D. Wasserman (Ed.), *Suicide: An unnecessary death* (pp. 27–38 2 ed.). Oxford University Press. 10.1093/med/9780198717393.003.0003

[cit0053] Wihlborg, J. , Edgren, G. , Johansson, A. , & Sivberg, B. (2017). Reflective and collaborative skills enhances ambulance nurses’ competence - A study based on qualitative analysis of professional experiences. *International Emergency Nursing* , *32* , 20–27. 10.1016/j.ienj.2016.06.002 27374021

[cit0054] Wiklund, L. , Lindholm, L. , & Lindström, U. A. (2002). Hermeneutics and narration: A way to deal with qualitative data. *Nursing Inquiry* , *9* (2), 114–125. 10.1046/j.1440-1800.2002.00132.x 12071912

[cit0055] World Health Organization . (2021). Suicide. World Health Organization. https://www.who.int/news-room/fact-sheets/detail/suicide.

